# Identification of survivin as a promising target for the immunotherapy of adult B-cell acute lymphoblastic leukemia

**DOI:** 10.18632/oncotarget.23380

**Published:** 2017-12-17

**Authors:** Laurie Freire Boullosa, Payalben Savaliya, Stephanie Bonney, Laurence Orchard, Hannah Wickenden, Cindy Lee, Evelien Smits, Alison H. Banham, Ken I. Mills, Kim Orchard, Barbara-Ann Guinn

**Affiliations:** ^1^ School of Life Sciences – Biomedical Science Subject Group, University of Hull, Hull, HU7 6RX, UK; ^2^ Centre for Oncological Research, University of Antwerp, 2610 Antwerp, Belgium; ^3^ Department of Life Sciences, University of Bedfordshire, Park Square, Luton, LU1 3JU, UK; ^4^ Cancer Sciences Unit, Somers Cancer Sciences Building, University of Southampton, Southampton SO16 6YD, UK; ^5^ Department of Haematology, University Hospital Southampton NHS Foundation Trust and University of Southampton, Southampton SO16 6YD, UK; ^6^ Nuffield Division of Clinical Laboratory Sciences, Radcliffe Department of Medicine, University of Oxford, John Radcliffe Hospital, Headington, Oxford OX3 9DU, UK; ^7^ Centre for Cancer Research and Cell Biology, Queens University Belfast, Belfast BT9 7AE, UK

**Keywords:** acute lymphocytic leukemia, antigen identification, immunotherapy, survivin, WT1

## Abstract

B-cell acute lymphoblastic leukemia (B-ALL) is a rare heterogeneous disease characterized by a block in lymphoid differentiation and a rapid clonal expansion of immature, non-functioning B cells. Adult B-ALL patients have a poor prognosis with less than 50% chance of survival after five years and a high relapse rate after allogeneic haematopoietic stem cell transplantation. Novel treatment approaches are required to improve the outcome for patients and the identification of B-ALL specific antigens are essential for the development of targeted immunotherapeutic treatments.

We examined twelve potential target antigens for the immunotherapy of adult B-ALL. RT-PCR indicated that only survivin and WT1 were expressed in B-ALL patient samples (7/11 and 6/11, respectively) but not normal donor control samples (0/8). Real-time quantitative (RQ)-PCR showed that survivin was the only antigen whose transcript exhibited significantly higher expression in the B-ALL samples (*n* = 10) compared with healthy controls (*n* = 4)(*p* = 0.015). Immunolabelling detected SSX2, SSX2IP, survivin and WT1 protein expression in all ten B-ALL samples examined, but survivin was not detectable in healthy volunteer samples. To determine whether these findings were supported by the analyses of a larger cohort of patient samples, we performed metadata analysis on an already published microarray dataset. We found that only survivin was significantly over-expressed in B-ALL patients (*n* = 215) compared to healthy B-cell controls (*n* = 12)(*p* = 0.013).

We have shown that survivin is frequently transcribed and translated in adult B-ALL, but not healthy donor samples, suggesting this may be a promising target patient group for survivin-mediated immunotherapy.

## INTRODUCTION

Acute lymphoblastic leukemia (ALL) is characterized by an excess of lymphoblasts of either the B- or T-lineage. If untreated the disease progresses rapidly and can be fatal within weeks to months. Adult patients with ALL who have had an allogeneic haematopoietic stem cell transplant (allo-HSCT) have an improved overall survival (OS) rate of 27–65% compared with 15–45% in the absence of allo-HSCT [1–3]. While the improvement in survival post-allogeneic HSCT may in part be due to the use of intensive chemotherapy and radiotherapy (such as total body irradiation) as conditioning, there does appear to be an increased survival advantage following HSCT using reduced intensity conditioning schedules in older patients and those with co-morbid risk factors [4, 5]. This suggests that post-transplant mechanisms, probably immunological in nature, play an important role in disease control with graft versus leukemia (GvL) effective in the eradication of residual disease. The ‘GvL effect’ has been demonstrated in other haematological malignancies, particularly chronic myeloid leukemia (CML), acute myeloid leukemia (AML) and myeloma, with the identification of probable immunological targets such as minor histocompatibility antigens [6], tumor specific antigens [7] and cancer-testis antigens (CTAs) [8].

A number of therapies have been, and are being, developed to target CD19, CD20, CD22 and/or CD52 on adult B-ALL patient blasts (recently reviewed in [9]). One of the most promising antibody therapies is blinatumab, which at the end of phase III clinical trials was shown to increase survival by months in patients with relapsed or refractory disease [10]. In addition, anti-CD19 chimeric antigen receptor-modified T cells (CAR-T-19) therapies have been shown to be able to induce complete remissions [11]. Such studies demonstrate the potential for immunotherapy to treat patients with B-ALL, with novel antigens providing additional targets that can be used to stimulate immune-mediate escape variant destruction.

Our own previous studies of adult B-ALL CD8+ T cells and their recognition of known leukemia antigens/epitopes therein [12] did not identify the same frequency/presence of antigen-specific T-cell populations as myeloid leukemia patients at disease diagnosis. The large numbers of affected lymphoblasts in the bone marrow of patients with adult B-ALL patients may lead to a lack of immune competent B and T cells in the periphery and may explain the general lack of tumor antigens identified to date. We examined the expression of a panel of cancer antigens in adult B-ALL to determine whether any would be promising targets for the immunotherapy of this difficult to treat disease.

## RESULTS

### Reverse transcription-polymerase chain reaction (RT-PCR) analysis of cell lines, patient samples and healthy donors

**Figure 1:**
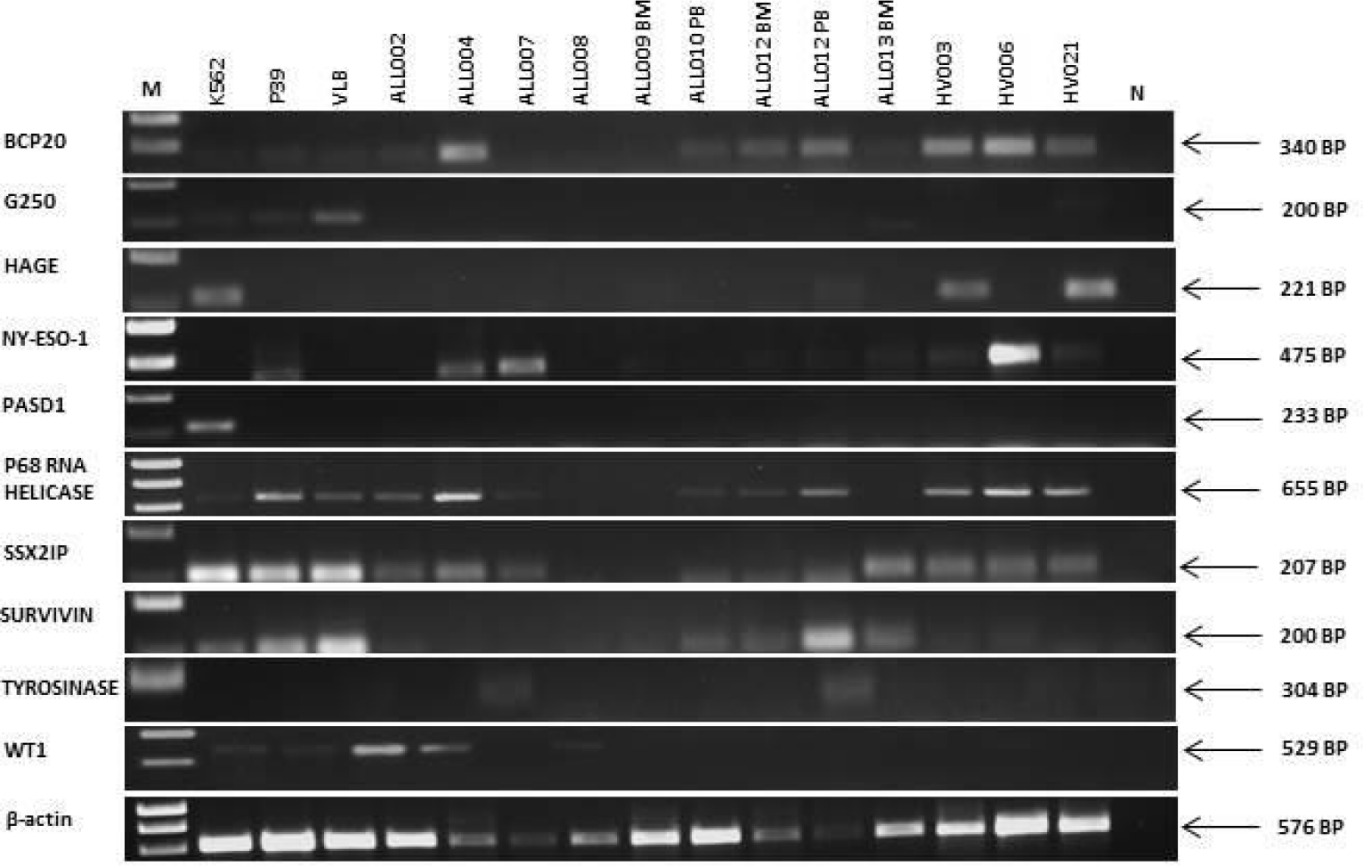
Expression of transcripts encoding leukemia antigens in cell lines, primary adult B-ALL patients and normal donors β-actin was used as a positive control for the ability of primers to amplify the cDNA. N was a no (cDNA) template control. Right hand side labels indicate expected product size and M indicates the location of the 1kB marker (HyperLadder I). RT-PCR data is representative of at least two independent experiments.

We examined the expression of twelve antigens (BCP-20, G250, HAGE, END, NY-ESO-1, PASD1, p68 RNA helicase, SSX2, SSX2IP, survivin, tyrosinase and WT1), identified as promising through a review of the literature, in human cancer cell lines to demonstrate consistency with previously published data and to optimise our assays (Supplementary Table 1). These results provided positive and negative controls for the expression of each antigen (Table 2A). We then examined the expression of the same twelve antigens in thirteen samples from eleven adult B-ALL patients (including twelve samples taken from patients prior to the start of any treatment) and eight healthy volunteers (Table 1). No suitable sample was available from ALL003 for RT-PCR analysis. RT-PCR analysis showed that two antigens were expressed in B-ALL patient samples (Table 2B) but not healthy donor samples. These were survivin (7/11 B-ALL patients) and *WT1* (6/11 B-ALL patients) with no detectable antigen expression in eight healthy volunteer samples (Figure 1; Table 2B). All other genes studied (BCP-20, END, G250, HAGE, NY-ESO-1, p68 RNA helicase, SSX2IP and tyrosinase) were detectable in patient samples and healthy volunteers, except PAS domain-containing protein 1 (PASD1) and SSX2 which were not detected in either. Due to limited sample availability we choose six of the antigens, that were differentially expressed in patients compared with normal controls (Survivin, WT1 and END) or of particular interest to our group (PASD1, SSX2, SSX2IP), for further investigation by qPCR.

**Table 1A:** Patient information

ID	Disease stage	Age (at sampling)	Sex
ALL001	Pre-treatment	NK	NK
ALL002	Follow-up	19	Male
ALL003	Pre-treatment	24	Male
ALL004	Diagnosis	22	Male
ALL005	Diagnosis	64	Male
ALL006	Pre-treatment	19	Male
ALL007	Diagnosis	19	Male
ALL008	Pre-treatment	19	Female
ALL009	Diagnosis	49	Male
ALL010	Diagnosis	19	Male
ALL011	Pre-treatment	18	Male
ALL012	Diagnosis	52	Female
ALL013	Pre-treatment	52	Male

NK, not known.

**Table 1B:** Healthy control (all PB)

ID	Age	Sex
HV003	19	Male
HV004	NK	NK
HV006	26	Female
HV007	19	Male
HV008	40	Female
HV016	26	Male
HV021	NK	Male
HV032	NK	NK
HV033	NK	NK

NK, not known.

Table 2RT-PCR expression of the ten leukemia antigens of interest in (a) human cancer cell lines (b) adult B-ALL patient samples and healthy volunteers

**(a):** 

Antigens/ Cell lines	β-actin	GAPDH	BCP-20	G250	HAGE	END	NY-ESO-1	PASD1	P68 RNA helicase	SSX2	SSX2IP	SURVIVIN	Tyrosinase	WT1
**697**	+	+	–	–	–	+	–	–	–	–	–	–	–	–
**ARH77**	+	+	+	–	–	+	–	–	–	–	+	–	–	–
**HeLa**	+	+	+	–	–	+	–	+	–	–	+	–	–	+
**H1299**	+	+	+	–	+	+	+	+	–	–	+	–	–	+
**Jurkats**	+	+	–	–	+	+	–	–	–	–	–	+	–	–
**K562**	+	+	+	+	+	+	–	+	+	+	+	+	–	+
**KG1**	+	+	–	–	–	+	–	–	–	–	–	+	–	–
**KYO-1**	+	+	+	–	–	+	–	+	–	–	–	–	–	–
**NB4**	+	+	–	–	–	+	–	+	–	–	–	–	–	+
**P39**	+	+	+	+	–	+	+	–	+	–	+	+	–	+
**SW480**	+	+	–	+	–	+	–	–	–	–	+	–	–	–
**U266**	+	+	+	–	–	+	–	–	–	–	+	–	–	+
**U937**	+	+	–	–	–	+	–	–	–	–	–	–	–	–
**VLB**	+	+	+	+	–	+	–	–	+	–	+	+	–/+	+
**Total**	**14/14**	**14/14**	**8/14**	**4/14**	**3/14**	**14/14**	**2/14**	**5/14**	**3/14**	**1/14**	**8/14**	**5/14**	**1/14**	**7/14**

**(b):** 

		Controls^†^		Antigens											
				β-actin	GAPDH	BCP-20	END	G250	HAGE	NY-ESO-1	PASD1	P68 RNA helicase	SSX2	SSX2IP	SURVIVIN	Tyrosinase	WT1
**Patient samples**	**ALL001**	+	+	+	+	–	+	+	–	+	–	–	–	–	+
	**ALL002**	+	+	+	+	–	–	–	–	+	–	+	+	–	+
	**ALL004**	+	+	–	+	–	–	+	–	+	–	+	–	+	–
	**ALL005**	+	+	+	–	–	–	+	–	+	–	–/+	+	–	+
	**ALL006**	+	+	+	+	+	+	+	–	+	–	+	+	–	+
	**ALL007**	+	+	–	+	–	–	+	–	+	–	+	–	–	+
	**ALL008**	+	+	–	+	–	–	–	–	–	–	–	–	–	–
	**ALL009BM**	+	+	–	+	–	–	+	–	–	–	–	+	–	–
	**ALL010BM**	+	+	+	+	+	+	+	–	+	–	+	+	+	–
	**ALL010PB**	+	+	+	+	–	–	+	–	+	–	+	+	–	–
	**ALL012BM**	+	+	+	+	–		+	–	+	–	+	+	+	–
	**ALL012PB**	+	+	–	+	–	+	+	–	+	–	+	+	–	–
	**ALL013BM**	+	+	–	+	+	–	+	–	–	–	+	+	–	+
	**Total‡**	**11/11**	**11/11**	**6/11**	**10/11**	**3/11**	**4/11**	**9/11**	**0/11**	**9/11**	**0/11**	**8/11**	**7/11**	**3/11**	**5/11**
**HV samples (all PB)**	**HV003**	+	+	+	–	–/+	+	+	–	+	–	+	–	+	–
	**HV004**	+	+	–	–		–	–	–	–	–	–	–	–	
	**HV006**	+	+	–	–	+	–	+	–	+	–	+	–	–	–
	**HV007**	+	+	–	–	–	–	–	–	–	–	–	–	–	–
	**HV016**	+	+	–	–	–	–	–	–	–	–	–	–	–	–
	**HV021**	+	+	+	–	–+	+	+	–	+	–	+	–	+	–
	**HV032**	+	+	–	–	–	–	–	–	–	–	–	–	–	–
	**HV033**	+	+	–	–	–	–	–	–	–	–	–	–	–	–
	**Total**	**8/8**	**8/8**	**2/8**	**0/8**	**3/8**	**2/8**	**3/8**	**0/8**	**3/8**	**0/8**	**3/8**	**0/8**	**3/8**	**0/8**

BM: bone marrow; HV: healthy volunteer; PB: peripheral blood. ^†^: controls for the integrity of the cDNA samples for PCR amplification; ^‡^number of patients with antigen positive samples (*n* = 11) encompassing the analysis of 13 samples.

### qPCR analysis of antigen expression in B-ALL and healthy donor samples

**Figure 2:**
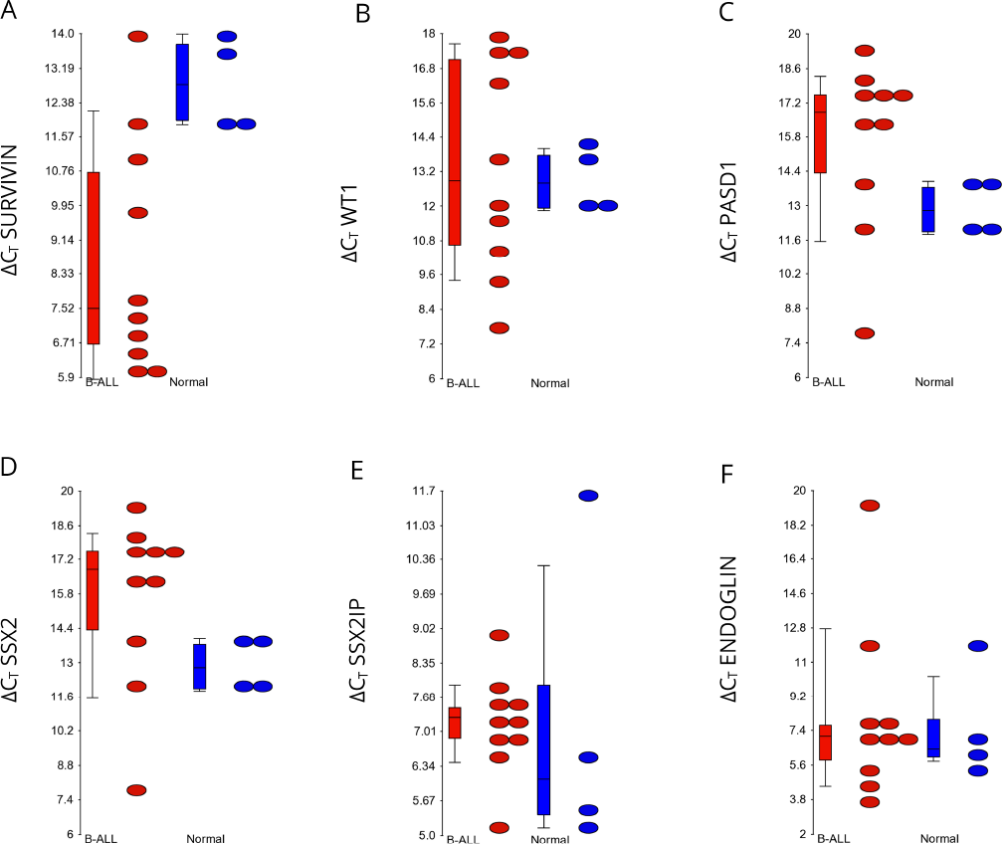
Relative expression of transcripts encoding each antigen in the B-ALL patients compared to the healthy volunteers Dots indicate the ΔC_T_ values, whereby the C_T_ of the endogenous control *GAPDH* is subtracted from the C_T_ of each gene. Genes that were not expressed were assigned a C_T_ value of 40. The higher the ΔC_T_ value, the less antigen was expressed. All ΔC_T_ values for antigens were lower level than the reference gene *GAPDH*. Streaked dots, representing patient sample ALL004, were outliers that do not represent antigen expression. *P*-values were determined using a two-way ANOVA test. Ns, not significant.

A two-way ANOVA test was used to determine whether there was a statistical difference between transcript expression of END, PASD1, SSX2, SSX2IP, Survivin and WT1, as determined by qPCR, in B-ALL patients (ALL001-8, 11 and 14) compared with healthy volunteers. Survivin had a significantly higher expression in seven of the ten B-ALL patients analysed, compared to healthy controls (*p* = 0.015) (Figure 2A). Its median ΔC_T_ value (7.19) in patients was much lower compared to the median ΔC_T_ value (12.81) in normal controls. *WT1* was expressed by three out of ten adult B-ALL patients (Figure 2B) however the median ΔC_T_ of B-ALL patients and normal controls, 12.88 and 12.81 respectively, were almost equal. Therefore, there was no significant difference detected by the two-way ANOVA test between these two groups.

Expression of *PASD1* and synovial sarcoma, X breakpoint 2 (*SSX2)* expression were not detected in any of the adult B-ALL patients or healthy volunteers (Figures 2C and 2D). Nine out of ten patients expressed *SSX2IP* (Figure 2E) while seven out of ten expressed END (Figure 2F). Although the expression of these genes were high, their transcripts were also found in three of five healthy volunteers.

### Immunolabelling of antigen expression in B-ALL using immunocytochemistry

**Figure 3:**
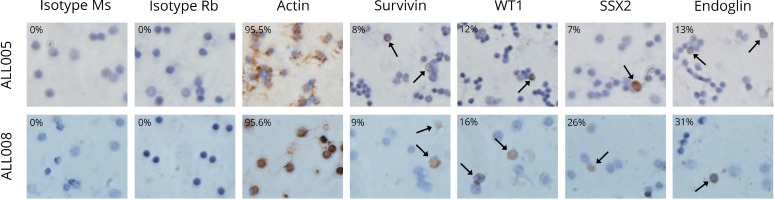
Immunolabelling of the key antigens of interest in two primary adult B-ALL patient samples Brown precipitate indicates the immunlabelling of test protein in the cell. Actin acted as the positive control for the assay. Two isotype control antibodies (Ms and Rb isotype) were used as negative controls to ensure there was minimal non-specific antibody binding to antigen. All pictures were taken at 400X magnification and were representative of at least two independent experiments.

The cell lines K562, OCI-LY3 and MDA-MB-231 were used to demonstrate the effectiveness of immunolabelling to detect the expression of END, PASD1, SSX2, SSX2IP, survivin, and WT1 (Table 3). Four out of five antigens had a cytoplasmic and nuclear localisation, while WT1 was only found in the cytoplasm of the K562 cells (Figure 3). The immunoreactivity score of both survivin and WT1 was moderate, while SSX2 and SSX2IP showed a weak labelling in K562 (Table 3). END was not expressed in the K562 cell line. OCI-LY-3 cell line was used as an extra control for the expression of PASD1 and showed high levels of PASD1 in the cytoplasm and near the cell membrane. END was moderately expressed on the surface of MDA-MB-231 cells grown on coverslips, confirming the findings of previous studies [13].

**Table 3:** Frequency of immunolabelling of the six antigens of interest in K562, OCI-LY-3 and MDA-MB-23 cells.

Staining intensity				Stained cells per microscopic image (%)			Immunoreactivity score			Subcellular localisation		
	K562	OCI-LY-3	MDA-MB-231	K562	OCI-LY-3	MDA-MB-231	K562	OCI-LY-3	MDA-MB-231	K562	OCI-LY-3	MDA-MB-231
Actin	4	4	4	185/203 (91)	563/569 (99)	53/53 (100)	364 (Very high)	396 (Very high)	400 (Very high)	Cytoplasm, nucleus	Cytoplasm, nucleus	Cytoplasm, nucleus
END	0	ND	3	0 (0)	ND	5/45 (11.1)	0 (Negative)	ND	33 (Moderate)	ND	ND	Surface
PASD1	2	3	ND	18/261 (6.9)	181/292 (62)	ND	14 (Weak)	186 (High)	ND	Cytoplasm, nucleus	Surface, cytoplasm	ND
SSX2	2	0	ND	23/255 (9)	0 (0)	ND	18 (Weak)	0 (Negative)	ND	Cytoplasm, nucleus	-	ND
SSX2IP	2	1	ND	17/195 (8.7)	45/241 (18.7)	ND	17 (Weak)	19 (Weak)	ND	Cytoplasm, nucleus	Surface	ND
Survivin	2.5	2.5	ND	71/190 (37.4)	67/364 (18.4)	ND	94 (Moderate)	46 (Moderate)	ND	Cytoplasm, nucleus	Cytoplasm, nucleus	ND
WT1	2.5	1	ND	36/236 (15.3)	50/352 (14.2)	ND	38 (Moderate)	14 (Weak)	ND	Cytoplasm	Cytoplasm	ND

Actin acted as positive control. Negative controls were cells only, two isotype and no primary antibody controls. Data was representative of two independent experiments. ND, not done.

Actin was used as a positive control for ICC immunolabelling and, as expected, all patients and healthy volunteers had a moderate (30–143) to high immunoreactivity score (144–228) (Table 4). Two isotype matched control antibodies (Supplementary Table 2) or no primary antibody controls were used as negative controls to determine whether there was any non-specific staining. All patient samples (*n* = 10) expressed moderate to high levels of each antigen (SSX2, SSX2IP, Survivin and WT-1) while survivin expression was not detected in the healthy volunteer samples. In contrast, one of six healthy volunteers expressed SSX2 at High levels, two of six healthy volunteers had detectable SSX2IP expression detectable at moderate levels, and WT1 at moderate levels. The ICC experiments were performed twice, with controls, due to limited samples being available, but the results were reproducible.

**Table 4:** Immunoreactivity score for the antigens SSX2, survivin, SSX2IP and WT1 as detected by ICC in B-ALL patient samples and healthy donors

Patient number	Cells only	Controls#	Isotype Rabbit	Actin	SSX2	SSX2IP	Survivin	WT1
ALL001	0	0	0	96	83.5 (mod)	89.5 (mod)	82.5 (mod)	94.5 (mod)
ALL002	0	0	0	300	196 (high)	99.5 (mod)	89.5 (mod)	200 (high)
ALL003	0	0	0	98.5	187 (high)	200 (high)	98 (mod)	92.5 (mod)
ALL004	0	0	0	94.5	91 (mod)	96 (mod)	95.5 (mod)	84.5 (mod)
ALL005	0	0	0	198	188 (high)	178 (high)	93.5 (mod)	95.5 (mod)
ALL006	0	-	0	200	200 (high)	198 (high)	95.5 (mod)	96 (mod)
ALL007	0	0	0	200	198 (high)	200 (high)	186 (high)	96 (mod)
ALL008	0	0	0	200	84 (mod)	91 (mod)	89.5 (mod)	79 (mod)
ALL009 (BM)	0	0	0	197	188 (high)	176 (high)	195 (high)	181 (high)
ALL009 (PB)	0	0	0	200	171 (high)	90.5 (mod)	175 (high)	196 (high)
ALL010 (BM)	0	0	0	99	93 (mod)	95 (mod)	89 (mod)	99 (mod)
ALL010 (PB)	0	0	0	97.5	181 (high)	200 (high)	100 (mod)	100 (mod)
Total	0/10	0/10	0/10	10/10	10/10	10/10	10/10	10/10
HV003	0	0	0	273	0	0	0	35.5 (mod)
HV004	0	0	0	198	151.5 (high)	38 (mod)	0	0
HV007	0	0	0	267	0	0	0	0
HV008	0	0	0	187	0	0	0	0
HV016	0	0	0	288	0	0	0	64.5 (mod)
HV021	0	0	0	189	0	0	0	0
Total	0/6	0/6	0/6	6/6	1/6	1/6	0/6	2/6

Actin acted as a positive control. ^**#**^The two isotype antibodies were used as negative controls or one isotype control and one test with no primary antibody. Immunoreactivity scores are as follows:- 0 = negative; 1–29:weak; 30–143: moderate (mod) and 144–228: high; >228: very high. Data is representative of at least two independent experiments.

### Gene expression analysis

Bioinformatic analysis of the publically available gene expression data set GSE38403 [14], indicated that survivin (*p* = 0.013) was significantly over-expressed in the B-ALL patient cohort (*n* = 215) compared to healthy B-cell controls (*n* = 12). Furthermore, of the twelve candidate genes investigated only p68 DNA helicase, SSX2IP, Survivin and WT1 showed significant differences in expression when compared across individual cytogenetic groups (Table 5). Elevated END or survivin expression was significantly associated with the t(9;21) translocation while p68 DNA helicase, SSX2IP, Survivin and WT1 expression were associated with different 11q23 /MLL abnormalities. We did examine whether there was a correlation between OS and event free survival (EFS) with the expression of each gene but none achieved significance and the closest to achieving significance was SSX2 interacting protein (SSX2IP) with an association with OS with a *p* value of 0.078.

**Table 5:** Microarray analysis indicating the significance of the association between the expression of each antigen and sample type, ALL compared with healthy pre B-cells and the presence or absence of cytogenetics abnormalities

	*p*-value							
Antigens	Sample type	Healthy preB cells vs. ALL	Cytogenetics abnormalities	NA vs. BCR/ABL^†^	NA vs. MLL/AF4^†^	NA vs. MLL_AF9^†^	NA vs. MLL_ENL^†^	NA vs. MLL_EPS15^†^
SSX2IP	NS	NS	0.0038	NS	0.032	0.018	0.043	NS
P68 RNA Helicase	NS	NS	0.0256	NS	NS	0.003	NS	NS
WT1	NS	NS	4.23E-11	NS	4.65E-06	0.026	0.0005	0.01
SURVIVIN	0.015	0.015	0.0035	0.003	NS	0.024	NS	0.023

Only those antigens that had a significant association between their expression and the clinical features analysed are shown.

NA: no abnormality; NS: not significant †: single cytogenetic abnormalities.

^**^ - highly significant.

^***^ - very highly significant.

## DISCUSSION

Most patients with adult B-ALL achieve first remission with conventional treatment however many relapse with high associated mortality. There is an acute need for therapies that can remove minimal residual disease and delay, if not prevent, relapse for these patients. To this end, we have investigated twelve known leukemia antigens for their expression in adult B-ALL.

Survivin and WT1 were the only antigens detected in patients but not healthy volunteers by RT-PCR and ICC, but only survivin had a statistically significant elevation in expression between the adult B-ALL cohort and healthy volunteer group by qPCR. This adds to a growing body of studies that have shown an association between survivin expression and ALL. Survivin is upregulated in a large number of solid tumors and haematological malignancies, including AML and ALL [15]. It acts as a dual regulator of both apoptosis and cell cycle progression, and is a member of the inhibitors of apoptosis proteins (IAP) family. Survivin plays a role in the cells’ escape from apoptotic pathways and is considered an important mechanism that facilitates leukaemogenesis and the resistance of tumors to chemotherapy. Survivin overexpression has been shown to initiate haematologic malignancies in transgenic mice [16] while its synthesis and degradation is controlled in a cell cycle-dependent course, with increasing transcription during G_1_ that peaks in G_2_-M. This supports its role in the regulation of the mitotic spindle checkpoint [17]. A study by Esh and colleagues showed that the knockdown of survivin mRNA via short-hairpin RNA or a locked antisense oligonucleotide reduced its gene expression, increased apoptosis in leukemia cell lines and accumulated the cells in the sub-G1 phase of the cell cycle [18]. In addition, silencing of the survivin gene in an ALL xenograft animal model improved chemotherapeutic responses while overexpression of the survivin gene has been associated with poor prognosis in paediatric ALL patients [18]. Mori and colleagues examined survivin expression in ALL patients using RT-PCR and found survivin expression in 11 of 16 ALL patients, but not in normal bone marrow (BM) cells [19]. Yang *et al.* also identified an elevation in vascular endothelial growth factor (*VEGF*) levels that coincided with survivin levels in 40 ALL patients by RT-PCR and western blotting [20].

Due to its limited expression in normal non-foetal tissues, survivin is a highly attractive immunotherapeutic target. When analysing five HLA-A2 positive adult ALL patient samples on a pMHC array [12] we did not detect any survivin-specific T cells that had bound to either of the HLA-A2 restricted survivin epitopes examined (survivin 5–11 or 96–104; [21]) even at a detection sensitivity of at least 0.02% of the CD8+ population. However other groups have found that survivin-specific T cell responses can be expanded in a number of pre-clinical and clinical settings, most recently a Phase II multi-epitope vaccine of five survivin peptides with adjuvant that resulted in the expansion of survivin-specific T cell responses in patients with solid cancers [22]. Although survivin was listed as one of the top 15 prioritised antigens by virtue of its therapeutic function, immunogenicity, specificity and oncogenicity among other features [23], it has been shown to be downregulated in both chronic myeloid leukemia (CML) [24] and AML-derived leukaemic stem cells (LSCs) [25]. Although this negatively impacts the value of survivin for the immunotherapy of myeloid leukemia, it’s epitopes have been characterised for immunotherapy (recently reviewed in [26]) and are likely to be useful in the generation of anti-tumor responses that may lead to epitope spreading and, at the least, could elongate the remission period and enable the administration of LSC-targeting treatments.

Our previous serological analysis of recombinant tumor cDNA expression libraries (SEREX) analysis of AML patient sera, identified *SSX2IP* and *PASD1*, as well as SSX2IP’s interacting partner, SSX2, as potential targets for the immunotherapy of myeloid leukemia [27]. Both PASD1 and SSX2 are CTAs that are expressed in cancer cells and immunologically protected sites. This restricted expression makes CTAs attractive targets for immunotherapy as their targeting should not lead to catastrophic auto-immune responses against healthy tissue. However, *PASD1* and *SSX2* transcripts were not detected in any of the adult B-ALL patients or healthy controls, although all of the adult B-ALL patient samples examined showed positive immunolabelling for SSX2 at moderate to high levels. This indicates that SSX2 protein expression may be worthy of further investigation in B-ALL patient samples, and our results suggest a lack of correlation between detectable *SSX2* transcription and the presence of SSX2 protein.

In contrast to our own previous studies [27], *SSX2IP* transcripts were found in patient samples and healthy control PB by RT-PCR, and qPCR, suggesting an improved sensitivity in our detection of *SSX2IP* transcripts in the last decade, as the RT-PCR primers and reagents remained unchanged. Surprisingly NY-ESO-1 transcripts were also found in B-ALL patient samples and normal donor PB samples by RT-PCR. Both findings require further investigation but may reflect the rapid proliferation and enhanced turnover of the white blood cells compared with many other healthy cell types or a technical error on our part, although we used multiple controls to ensure we did not have any contaminating gDNA in our cDNA samples. In addition we used previously published primers with our own validated techniques. BCR/ABL, a hallmark of CML has also been found in 10–30% of tested healthy adults increasing in prevalence with donor age [28]. The Philadelphia chromosome translocation product has been shown to be essential for the development of CML yet remains present in healthy donors without doing so, suggesting an, as yet undefined, requirement for additional events to achieve full transformation to the malignant phenotype. Ismail *et al.* [29] suggested this occurrence was due to an accumulation of DNA damage with age and it may be a feature of the rapidly proliferating, high turnover “normal” white blood cells.

Another of the potential immunotherapeutic targets for B-ALL that we investigated was the LAA WT1. Inoue *et al.* demonstrated consistently increased *WT1* expression levels in most myeloid and lymphoid acute leukemias via RT-PCR [30]. These results were confirmed by Cilloni *et al*, who detected WT1 overexpression in all 48 ALL samples at diagnosis (BM and PB) when using qPCR [31]. They showed that *WT1* expression was detectable, but that *WT1* transcript levels in normal BM and PB were extremely low and often below the qPCR detection limit. Therefore, *WT1* is a promising marker to discriminate between normal and leukaemic haematopoiesis, and effective in establishing the presence, persistence and/or reappearance of leukaemic blasts for diagnosis or detection of minimal residual disease (MRD). Our qPCR results demonstrated *WT1* mRNA expression in three out of the ten adult B-ALL patients, but none of the healthy volunteers.

In summary, our study has demonstrated the value of pursuing survivin as a target for the immunotherapy of adult B-ALL, through our demonstration of its transcription and translation as early as disease diagnosis. This is a rare disease with high associated mortality. The fact that most patients can achieve first remission provides a time point during which residual tumor cells may be targeted by immunotherapy. The reduction or, ideally, removal of MRD could provide an opportunity to extend, if not prevent, relapse benefitting patient survival. A number of clinical trials are underway that target survivin including those using immunotherapy protocols or survivin-inhibitors. Our study has identified a patient group who would likely benefit from their application and warrants further investigation.

## MATERIALS AND METHODS

### Cell lines and patient samples

Human cancer cell lines were used to measure the expression of the antigens and optimise assays. All were obtained from ATCC (Sigma-Aldrich Co. Ltd) and grown in RPMI 1640 or DMEM media (Sigma-Aldrich Company Ltd., Dorset, U.K.) containing 10% foetal bovine serum (FBS) and 1% penicillin and streptomycin (both Thermo Fisher Scientific, Leicestershire, UK), in a humidified incubator at 37°C with 5% CO_2._ K562 was positive for the expression of most antigens examined (Supplementary Table 2) as described previously.

16 samples were collected from 14 adult B-ALL patients at various treatment time points, but predominantly diagnosis and pre-treatment (Table 1A), from the Departments of Haematology at University Hospital Southampton NHS FT, Portsmouth Hospitals NHS Trust and the Royal Devon and Exeter Foundation Trust following informed consent and local ethical approval (REC 07/H0606/88). Leukaemic blasts and mononuclear cells were isolated from PB and/or BM in EDTA. White blood cells were also isolated from age and sex-matched normal donor PB following informed consent and local ethical approval (LREC 228/02/T).

### Identification of antigens for study in B-ALL

Due to a lack of known antigens that can act as targets for the immunotherapy of B-ALL, we identified a list of antigens of potential interest for further study. We had previously identified PASD1, SSX2IP and HAGE expression in presentation AML [27, 32] and examined presentation acute leukemia patients for T cells that recognized epitopes within G250, NY-ESO-1, tyrosinase, p68 RNA helicase, WT1 and survivin [12]. We examined SSX2 because of its known interaction with SSX2IP [33], BCP-20 based on its expression in solid tumors [34] and END based on its detection in paediatric leukemia and association with patient outcome [35].

### RT-PCR analysis of antigen expression in patient and normal donor samples

To evaluate the expression of the most promising antigens in normal and malignant tissues we isolated RNA from BM and PB samples using QIAGEN RNeasy kit (QIAGEN Ltd.). mRNA was DNase I treated (Roche Products Ltd, Herts, U.K.), cleaned using a RNeasy kit (Qiagen), checked on a 1% agarose-TBE gel and quantified using a spectrophotometer. We prepared cDNA using the MBI Fermentas RevertAid First Strand cDNA synthesis kit (MBI Fermentas Ltd, Helena BioSciences Ltd, Sunderland, U.K.), utilising the random hexamer primers.

To test the fidelity of the cDNA samples, each cDNA was subjected to β-actin PCR. All PCR amplifications were performed on 200 ng of cDNA, mRNA (to show no gDNA was present in the mRNA samples) or gDNA (to demonstrate that the amplification of gDNA led to a product of a larger size than cDNA). We used 12.5 µl ReadyMix Taq PCR reaction mix (Sigma) and 1.5 mM MgCl_2_ in a final volume of 25 µl and primers were used as described previously:- *BCP-20* [36], *G250* [37], *HAGE* [32], *NY-ESO-1* [38], *PASD1* [27], p68 RNA helicase [39], SSX2 [40], *SSX2IP* [27], tyrosinase [41] and *WT1* [42]. To detect survivin we used the forward: 5-GATGACGACCCCATAGAGGAAC-3′ and reverse: 5′- GGGTTAATTCTTCAAACTGCTTCT-3′ primers [43]. Two primers were designed using the Primer3 Output MIT educational tool (www.genome.wi.mit.edu) to flank introns and were assessed for specificity using BLAST web based programmes. These were β-actin forward 5′-CATGGATGATGATATCGCCGC-3′ and reverse 5′-CCATCACGCCCTGGTGC-3′ and END forward 5′-CCCTTCTCTAAGGAAGCGCA-3′ and reverse 5′- AGTTGCTGTCCGAAGGATGG-3′. PCR products were electrophoresed on 1% TBE-agarose gels and assessed following staining with 10 µg/ml ethidium bromide.

### Sequencing of PCR products

After gel electrophoresis of the PCR products and image capture, bands were excised from the agarose gel and placed in 1.5 ml sterile eppendorf tube. These products were extracted from the gel bands using the PCR gel extraction kit (Qiagen). Where available, products from three independent PCR reactions on the same template were sent for Sanger sequencing to the DNA sequencing facility at the University of Cambridge. We analysed each sequence using Finch TV software and BLASTN to compare similarity between the PCR products and their target cDNA sequences.

### QPCR analysis

QPCR was performed using SYBR Green technology with the QuantiTect Primer Assays and QuantiNova SYBR Green PCR kit (all Qiagen), to investigate the relative expression of six TAAs in ten adult B-ALL samples (ALL001-8, 11 and 14), as well as *GAPDH* as a control for sample loading and the quality of the cDNA. Each primer was tested on at least one human cancer cell line that was known to express the antigen of interest (Supplementary Table 2), based on previously published studies. To control for contamination within the qPCR reagents, a no cDNA control was included on every qPCR plate whereby cDNA was replaced by RNase-free H_2_O. In addition, each sample was plated in triplicate on the 96-well qPCR plate (Applied Biosystems, USA) to identify any outliers in the dataset. The reaction volumes were 10 µL 2X QuantiNova SYBR green PCR MM, 0.1 µL ROX reference dye, 2 µL primer assay and 6.9 µL RNase-free H_2_O, making a total volume of 19 µL added to each well in the 96-well plate.

The thermocycler (StepOne Plus Real-Time PCR system, Applied Biosystems) ran a heating step for 2 minutes at 95°C, then 40 cycles were run, whereby each cycle was set to denature for 5 s at 95°C, and to anneal plus extend primers in a combined step for 10 s at 60°C. This was immediately followed by a melt curve stage of 15 s at 95°C, 1 min at 60°C and 15 s at 95°C, to verify the specificity of the amplification, e.g. no non-specific primer dimer formation. Data was compared using StepOne software v2.0 (Applied Biosystems) and the comparative C_T_ method [44]. When comparing antigen expression in B-ALL to healthy controls, the results were normalised with the *GAPDH* reference gene (ΔC_T_ = C_T_ antigen–C_T_ GAPDH). All qPCR data were analysed with a two-way ANOVA test for pairwise comparisons, using Partek Genomic Software (Partek Inc., USA).

### Immunocytochemistry

Leukocytes were isolated following a 30min incubation of PB and/or BM samples with red cell lysis buffer (155 mM NH_4_Cl, 10 mM KHCO_3_, 0.1 mM EDTA), after which leukocytes were pelleted by centrifugation for 10 min at 800 g. Leukocytes or cells from lines were resuspended in PBS at 5 × 10^6^/ml, with 10 ul cells spotted at each of two sites on microscope glass slides. Slides were double wrapped in saran wrap and stored at –20^o^C until required for use. Immunocytochemistry was performed as described previously [45] using antibodies as detailed in Supplementary Table 1.

Due to the cellular localisation of END on the cell surface, the MDA-MB-231 cell line was grown to 50–70% confluence on glass coverslips. Then, the culture medium from each well was aspirated and cover slips were rinsed in PBS. The coverslips were air dried for 4–6 h, wrapped in saran wrap and stored in –20°C freezer. Actin was used as a positive control for the successful performance of ICC while isotype, no primary and no secondary antibody immunolabelling acted as negative controls, used to detect non-specific staining. Lillies-Mayer Haematoxylin was used as a countertstain.

Staining intensities were scored according to a five-tiered scale described originally by [46] as follows:- 0: no staining; 1: background; 2: weak staining; 3: moderate staining; 4: strong staining. The percentage of positively stained cells was based on the cell count of stained cells per microscopic view, and represented on a five-tiered scale (0: 0%; 1: 1–10%; 2: 11–50%; 3: 51–80%; 4: > 80%). The final immunoreactivity score was obtained by multiplication of the percentage of positive stained cells per microscopic view by the value for staining intensity (0 = negative, 1–29 = weak, 30–143 = moderate, 144–228 = high and > 228 = very high staining) [47].

### Gene expression analysis

To determine the relative expression of the antigens of interest in a larger cohort of adult B-ALL samples and healthy controls, we performed metadata analysis on a publically available microarray expression data (GSE38403) [14] which utilised 215 adult B-cell ALL and 12 normal pre-B samples. The CEL files were downloaded and imported in the Partek Genomic Suite, normalised using RMA, and subjected to ANOVA analysis which was filtered on significance for the generation of the gene lists.

## SUPPLEMENTARY MATERIALS TABLES



## Abbreviations

AML: acute myeloid leukemia
B-ALL: B-cell acute lymphocytic leukemia
BM: bone marrow
CML: chronic myeloid leukemia
CTA: cancer-testis antigens
GvL: graft versus leukemia
LAA: leukemia associated antigens
LSC: leukaemic stem cells
MRD: minimal residual disease
OS: overall survival
PASD1: PAS domain-containing protein 1
PB: peripheral blood
RT-PCR: reverse transcription – polymerase chain reaction
RQ: real-time quantitative
SSX2: synovial sarcoma, X breakpoint 2
SSX2IP: SSX2 interacting partner
WT1: Wilms tumor-1

## References

[1] Fielding AK, Rowe JM, Richards SM, Buck G, Moorman AV, Durrant IJ, Marks DI, McMillan AK, Litzow MR, Lazarus HM, Foroni L, Dewald G, Franklin IM (2009). Prospective outcome data on 267 unselected adult patients with Philadelphia chromosome-positive acute lymphoblastic leukemia confirms superiority of allogeneic transplantation over chemotherapy in the pre-imatinib era: results from the International ALL Trial MRC UKALLXII/ECOG2993. Blood.

[2] Goldstone AH, Richards SM, Lazarus HM, Tallman MS, Buck G, Fielding AK, Burnett AK, Chopra R, Wiernik PH, Foroni L, Paietta E, Litzow MR, Marks DI (2008). In adults with standard-risk acute lymphoblastic leukemia, the greatest benefit is achieved from a matched sibling allogeneic transplantation in first complete remission, and an autologous transplantation is less effective than conventional consolidation/maintenance chemotherapy in all patients: final results of the International ALL Trial (MRC UKALL XII/ECOG E2993). Blood.

[3] Moorman AV, Harrison CJ, Buck GA, Richards SM, Secker-Walker LM, Martineau M, Vance GH, Cherry AM, Higgins RR, Fielding AK, Foroni L, Paietta E, Tallman MS (2007). Karyotype is an independent prognostic factor in adult acute lymphoblastic leukemia (ALL): analysis of cytogenetic data from patients treated on the Medical Research Council (MRC) UKALLXII/Eastern Cooperative Oncology Group (ECOG) 2993 trial. Blood.

[4] Cho BS, Min CK, Eom KS, Kim YJ, Kim HJ, Lee S, Cho SG, Kim DW, Lee JW, Min WS, Kim CC (2009). Feasibility of NIH consensus criteria for chronic graft-versus-host disease. Leukemia.

[5] Stein AS, Palmer JM, O’Donnell MR, Kogut NM, Spielberger RT, Slovak ML, Tsai NC, Senitzer D, Snyder DS, Thomas SH, Forman SJ (2009). Reduced-intensity conditioning followed by peripheral blood stem cell transplantation for adult patients with high-risk acute lymphoblastic leukemia. Biol Blood Marrow Transplant.

[6] Feng X, Hui KM, Younes HM, Brickner AG (2008). Targeting minor histocompatibility antigens in graft versus tumor or graft versus leukemia responses. Trends Immunol.

[7] Yong AS, Keyvanfar K, Eniafe R, Savani BN, Rezvani K, Sloand EM, Goldman JM, Barrett AJ (2008). Hematopoietic stem cells and progenitors of chronic myeloid leukemia express leukemia-associated antigens: implications for the graft-versus-leukemia effect and peptide vaccine-based immunotherapy. Leukemia.

[8] Atanackovic D, Arfsten J, Cao Y, Gnjatic S, Schnieders F, Bartels K, Schilling G, Faltz C, Wolschke C, Dierlamm J, Ritter G, Eiermann T, Hossfeld DK (2007). Cancer-testis antigens are commonly expressed in multiple myeloma and induce systemic immunity following allogeneic stem cell transplantation. Blood.

[9] Wei G, Wang J, Huang H, Zhao Y (2017). Novel immunotherapies for adult patients with B-lineage acute lymphoblastic leukemia. J Hematol Oncol.

[10] Kantarjian HM, DeAngelo DJ, Advani AS, Stelljes M, Kebriaei P, Cassaday RD, Merchant AA, Fujishima N, Uchida T, Calbacho M, Ejduk AA, O’Brien SM, Jabbour EJ (2017). Hepatic adverse event profile of inotuzumab ozogamicin in adult patients with relapsed or refractory acute lymphoblastic leukemia: results from the open-label, randomised, phase 3 INO-VATE study. Lancet Haematol.

[11] Maude SL, Frey N, Shaw PA, Aplenc R, Barrett DM, Bunin NJ, Chew A, Gonzalez VE, Zheng Z, Lacey SF, Mahnke YD, Melenhorst JJ, Rheingold SR (2014). Chimeric antigen receptor T cells for sustained remissions in leukemia. N Engl J Med.

[12] Brooks SE, Bonney SA, Lee C, Publicover A, Khan G, Smits EL, Sigurdardottir D, Arno M, Li D, Mills KI, Pulford K, Banham AH, van Tendeloo V (2015). Application of the pMHC Array to Characterise Tumor Antigen Specific T Cell Populations in Leukemia Patients at Disease Diagnosis. PLoS One.

[13] Oxmann D, Held-Feindt J, Stark AM, Hattermann K, Yoneda T, Mentlein R (2008). Endoglin expression in metastatic breast cancer cells enhances their invasive phenotype. Oncogene.

[14] Geng H, Brennan S, Milne TA, Chen WY, Li Y, Hurtz C, Kweon SM, Zickl L, Shojaee S, Neuberg D, Huang C, Biswas D, Xin Y (2012). Integrative epigenomic analysis identifies biomarkers and therapeutic targets in adult B-acute lymphoblastic leukemia. Cancer Discov.

[15] Adida C, Recher C, Raffoux E, Daniel MT, Taksin AL, Rousselot P, Sigaux F, Degos L, Altieri DC, Dombret H (2000). Expression and prognostic significance of survivin in *de novo* acute myeloid leukemia. Br J Haematol.

[16] Small S, Keerthivasan G, Huang Z, Gurbuxani S, Crispino JD (2010). Overexpression of survivin initiates hematologic malignancies *in vivo*. Leukemia.

[17] Mita AC, Mita MM, Nawrocki ST, Giles FJ (2008). Survivin: Key Regulator of Mitosis and Apoptosis and Novel Target for Cancer Therapeutics. Clin Cancer Res.

[18] Esh AM, Atfy M, Azizi NA, El Naggar MM, Khalil EE, Sherief L (2011). Prognostic Significance of Survivin in Pediatric Acute Lymphoblastic Leukemia. Ind J Hematol Blood Transf.

[19] Mori A, Wada H, Nishimura Y, Okamoto T, Takemoto Y, Kakishita E (2002). Expression of the Antiapoptosis Gene Survivin in Human Leukemia. Int J Hematol.

[20] Yang M, Liu Y, Lu S, Wang Z, Wang RAN, Zi Y, Li J (2013). Analysis of the expression levels of survivin and VEGF in patients with acute lymphoblastic leukemia. Exp Therapeutic Med.

[21] Schmitz M, Diestelkoetter P, Weigle B, Schmachtenberg F, Stevanovic S, Ockert D, Rammensee HG, Rieber EP (2000). Generation of survivin-specific CD8+ T effector cells by dendritic cells pulsed with protein or selected peptides. Cancer Res.

[22] Lennerz V, Gross S, Gallerani E, Sessa C, Mach N, Boehm S, Hess D, von Boehmer L, Knuth A, Ochsenbein AF, Gnad-Vogt U, Zieschang J, Forssmann U (2014). Immunologic response to the survivin-derived multi-epitope vaccine EMD640744 in patients with advanced solid tumors. Cancer Immunol Immunother.

[23] Cheever MA, Allison JP, Ferris AS, Finn OJ, Hastings BM, Hecht TT, Mellman I, Prindiville SA, Viner JL, Weiner LM, Matrisian LM (2009). The prioritization of cancer antigens: a national cancer institute pilot project for the acceleration of translational research. Clin Cancer Res.

[24] Gerber JM, Qin L, Kowalski J, Smith BD, Griffin CA, Vala MS, Collector MI, Perkins B, Zahurak M, Matsui W, Gocke CD, Sharkis SJ, Levitsky HI (2011). Characterization of chronic myeloid leukemia stem cells. Am J Hematol.

[25] Gal H, Amariglio N, Trakhtenbrot L, Jacob-Hirsh J, Margalit O, Avigdor A, Nagler A, Tavor S, Ein-Dor L, Lapidot T, Domany E, Rechavi G, Givol D (2006). Gene expression profiles of AML derived stem cells; similarity to hematopoietic stem cells. Leukemia.

[26] Garg H, Suri P, Gupta JC, Talwar GP, Dubey S (2016). Survivin: a unique target for tumor therapy. Cancer Cell Int.

[27] Guinn BA, Bland EA, Lodi U, Liggins AP, Tobal K, Petters S, Wells JW, Banham AH, Mufti GJ (2005). Humoral detection of leukemia-associated antigens in presentation acute myeloid leukemia. Biochem Biophys Res Commun.

[28] Biernaux C, Loos M, Sels A, Huez G, Stryckmans P (1995). Detection of major bcr-abl gene expression at a very low level in blood cells of some healthy individuals. Blood.

[29] Ismail SI, Naffa RG, Yousef AM, Ghanim MT (2014). Incidence of bcrabl fusion transcripts in healthy individuals. Mol Med Rep.

[30] Inoue K, Ogawa H, Yamagami T, Soma T, Tani Y, Tatekawa T, Oji Y, Tamaki H, Kyo T, Dohy H, Hiraoka A, Masaoka T, Kishimoto T (1996). Long-term follow-up of minimal residual disease in leukemia patients by monitoring WT1 (Wilms tumor gene) expression levels. Blood.

[31] Cilloni D, Gottardi E, De Micheli D, Serra A, Volpe G, Messa F, Rege-Cambrin G, Guerrasio A, Divona M, Lo Coco F, Saglio G (2002). Quantitative assessment of WT1 expression by real time quantitative PCR may be a useful tool for monitoring minimal residual disease in acute leukemia patients. Leukemia.

[32] Adams SP, Sahota SS, Mijovic A, Czepulkowski B, Padua RA, Mufti GJ, Guinn BA (2002). Frequent expression of HAGE in presentation chronic myeloid leukemias. Leukemia.

[33] de Bruijn DR, dos Santos NR, Kater-Baats E, Thijssen J, van den Berk L, Stap J, Balemans M, Schepens M, Merkx G, van Kessel AG (2002). The cancer-related protein SSX2 interacts with the human homologue of a Ras-like GTPase interactor, RAB3IP, and a novel nuclear protein, SSX2IP. Genes Chromosomes Cancer.

[34] Song MH, Ha JC, Lee SM, Park YM, Lee SY (2011). Identification of BCP-20 (FBXO39) as a cancer/testis antigen from colon cancer patients by SEREX. Biochem Biophys Res Commun.

[35] Catchpoole D, Lail A, Guo D, Chen QR, Khan J (2007). Gene expression profiles that segregate patients with childhood acute lymphoblastic leukemia: an independent validation study identifies that endoglin associates with patient outcome. Leuk Res.

[36] Boncheva V (2013). The identification of tumor antigens recognized by patients with Duke’s B (Stage II) reactive colorectal cancers using SEREX. PhD Thesis.

[37] Liu J, Fang L, Cheng Q, Li L, Su C, Zhang B, Pei D, Yang J, Li W, Zheng J (2012). Effects of G250 promoter controlled conditionally replicative adenovirus expressing Ki67-siRNA on renal cancer cell. Cancer Sci.

[38] Ries J, Mollaoglu N, Vairaktaris E, Neukam FW, Nkenke E (2009). Diagnostic and therapeutic relevance of NY-ESO-1 expression in oral squamous cell carcinoma. Anticancer Res.

[39] Clark EL, Coulson A, Dalgliesh C, Rajan P, Nicol SM, Fleming S, Heer R, Gaughan L, Leung HY, Elliott DJ, Fuller-Pace FV, Robson CN (2008). The RNA helicase p68 is a novel androgen receptor coactivator involved in splicing and is overexpressed in prostate cancer. Cancer Res.

[40] Khan G (2016). Characterisation of the Expression of Tumor Antigens and Biomarkers in Myeloid Leukemia and Ovarian Cancer.

[41] Abrahamsen HN, Sorensen BS, Nexo E, Hamilton-Dutoit SJ, Larsen J, Steiniche T (2005). Pathologic assessment of melanoma sentinel nodes: a role for molecular analysis using quantitative real-time reverse transcription-PCR for MART-1 and tyrosinase messenger RNA.. Clin Cancer Res.

[42] Nagel H, Laskawi R, Eiffert H, Schlott T (2003). Analysis of the tumor suppressor genes, FHIT and WT-1, and the tumor rejection genes, BAGE, GAGE-1/2, HAGE, MAGE-1, and MAGE-3, in benign and malignant neoplasms of the salivary glands. Mol Pathol.

[43] Johnen G, Gawrych K, Bontrup H, Pesch B, Taeger D, Banek S, Kluckert M, Wellhausser H, Eberle F, Nasterlack M, Leng G, Stenzl A, Bruning T (2012). Performance of survivin mRNA as a biomarker for bladder cancer in the prospective study UroScreen. PLoS One.

[44] Livak KJ, Schmittgen TD (2001). Analysis of relative gene expression data using real-time quantitative PCR and the 2(-Delta Delta C(T)) Method. Methods.

[45] Khan G, Brooks SE, Mills KI, Guinn BA (2015). Infrequent Expression of the Cancer-Testis Antigen, PASD1, in Ovarian Cancer. Biomark Cancer.

[46] Biesterfeld S, Veuskens U, Schmitz FJ, Amo-Takyi B, Bocking A (1996). Interobserver reproducibility of immunocytochemical estrogen- and progesterone receptor status assessment in breast cancer. Anticancer Res.

[47] Deng Z, Hasegawa M, Aoki K, Matayoshi S, Kiyuna A, Yamashita Y, Uehara T, Agena S, Maeda H, Xie M, Suzuki M (2014). A comprehensive evaluation of human papillomavirus positive status and p16(INK4a) overexpression as a prognostic biomarker in head and neck squamous cell carcinoma. Int J Oncol.

[48] Alvarado CS, Kim T, Ragab AH (1982). Acute lymphocytic leukemia in childhood. J Med Assoc Ga.

[49] Rokhlin OW, Cohen MB, Kubagawa H, Letarte M, Cooper MD (1995). Differential expression of endoglin on fetal and adult hematopoietic cells in human bone marrow. J Immunol.

[50] Wiels J, Lenoir GM, Fellous M, Lipinski M, Salomon JC, Tetaud C, Tursz T (1982). A monoclonal antibody with anti-Burkitt lymphoma specificity. I. Analysis of human haematopoietic and lymphoid cell lines. Int J Cancer.

[51] Scherer WF, Syverton JT, Gey GO (1953). Studies on the propagation *in vitro* of poliomyelitis viruses. IV. Viral multiplication in a stable strain of human malignant epithelial cells (strain HeLa) derived from an epidermoid carcinoma of the cervix. J Exp Med.

[52] Lee M, Draoui M, Zia F, Gazdar A, Oie H, Bepler G, Bellot F, Tarr C, Kris R, Moody TW (1992). Epidermal growth factor receptor monoclonal antibodies inhibit the growth of lung cancer cell lines. J Natl Cancer Inst Monogr.

[53] Hardwick N, Buchan S, Ingram W, Khan G, Vittes G, Rice J, Pulford K, Mufti G, Stevenson F, Guinn BA (2013). An analogue peptide from the Cancer/Testis antigen PASD1 induces CD8+ T cell responses against naturally processed peptide. Cancer Immun.

[54] Lozzio CB, Lozzio BB (1975). Human chronic myelogenous leukemia cell-line with positive Philadelphia chromosome. Blood.

[55] Denniss FA, Breslin A, Ingram W, Hardwick NR, Mufti GJ, Guinn BA (2007). The leukemia-associated antigen, SSX2IP, is expressed during mitosis on the surface of myeloid leukemia cells. Br J Haematol.

[56] dos Santos NR, Torensma R, de Vries TJ, Schreurs MWJ, de Bruijn DRH, Kater-Baats E, Ruiter DJ, Adema GJ, van Muijen GNP, van Kessel AG (2000). Heterogeneous Expression of the SSX Cancer/Testis Antigens in Human Melanoma Lesions and Cell Lines. Cancer Research.

[57] Inoue K, Sugiyama H, Ogawa H, Nakagawa M, Yamagami T, Miwa H, Kita K, Hiraoka A, Masaoka T, Nasu K (1994). WT1 as a new prognostic factor and a new marker for the detection of minimal residual disease in acute leukemia. Blood.

[58] Schmidt SM, Schag K, Müller MR, Weck MM, Appel S, Kanz L, Grünebach F, Brossart P (2003). Survivin is a shared tumor-associated antigen expressed in a broad variety of malignancies and recognized by specific cytotoxic T cells. Blood.

[59] Koeffler HP, Billing R, Lusis AJ, Sparkes R, Golde DW (1980). An undifferentiated variant derived from the human acute myelogenous leukemia cell line (KG-1). Blood.

[60] Ohkubo T, Kamamoto T, Kita K, Hiraoka A, Yoshida Y, Uchino H (1985). A novel Ph1 chromosome positive cell line established from a patient with chronic myelogenous leukemia in blastic crisis. Leuk Res.

[61] Yoneda T, Williams PJ, Hiraga T, Niewolna M, Nishimura R (2001). A bone-seeking clone exhibits different biological properties from the MDA-MB-231 parental human breast cancer cells and a brain-seeking clone *in vivo* and *in vitro*. J Bone Miner Res.

[62] Lanotte M, Martin-Thouvenin V, Najman S, Balerini P, Valensi F, Berger R (1991). NB4, a maturation inducible cell line with t(15;17) marker isolated from a human acute promyelocytic leukemia (M3). Blood.

[63] Xue J, Lin MF (2005). [Factors regulating expression of antiapoptosis gene survivin]. [Article in Chinese]. Zhongguo Shi Yan Xue Ye Xue Za Zhi.

[64] Chang H, Benchimol S, Minden MD, Messner HA (1994). Alterations of p53 and c-myc in the clonal evolution of malignant lymphoma. Blood.

[65] Cooper CD, Liggins AP, Ait-Tahar K, Roncador G, Banham AH, Pulford K (2006). PASD1, a DLBCL-associated cancer testis antigen and candidate for lymphoma immunotherapy. Leukemia.

[66] Nagai M, Seki S, Kitahara T, Abe T, Minato K, Watanabe S, Shimoyama M (1984). A novel human myelomonocytoid cell line, P39/Tsugane, derived from overt leukemia following myelodysplastic syndrome. Gan.

[67] Leibovitz A, Stinson JC, McCombs WB, McCoy CE, Mazur KC, Mabry ND (1976). Classification of human colorectal adenocarcinoma cell lines. Cancer Res.

[68] Koesters R, Linnebacher M, Coy JF, Germann A, Schwitalle Y, Findeisen P, von Knebel Doeberitz M (2004). WT1 is a tumor-associated antigen in colon cancer that can be recognized by *in vitro* stimulated cytotoxic T cells. Int J Cancer.

[69] Liggins AP, Brown PJ, Asker K, Pulford K, Banham AH (2004). A novel diffuse large B-cell lymphoma-associated cancer testis antigen encoding a PAS domain protein. Br J Cancer.

[70] Nilsson K, Bennich H, Johansson SG, Ponten J (1970). Established immunoglobulin producing myeloma (IgE) and lymphoblastoid (IgG) cell lines from an IgE myeloma patient. Clin Exp Immunol.

[71] Beck WT, Mueller TJ, Tanzer LR (1979). Altered surface membrane glycoproteins in Vinca alkaloid-resistant human leukemic lymphoblasts. Cancer Res.

[72] Szemes M, Dallosso AR, Melegh Z, Curry T, Li Y, Rivers C, Uney J, Magdefrau AS, Schwiderski K, Park JH, Brown KW, Shandilya J, Roberts SG (2013). Control of epigenetic states by WT1 via regulation of *de novo* DNA methyltransferase 3A. Hum Mol Genet.

[73] Wang T, Liu Z, Zhang Z, Tang S, Yue M, Feng S, Hu M, Xuan L, Chen Y (2017). Evaluation of antitumor activity of survivin short interfering RNA delivered by lipid nanoparticles in colon cancer *in vitro* and *in vivo*. Oncol Lett.

