# Supplementary material for: Identification of survivin as a promising target for the immunotherapy of adult B-cell acute lymphoblastic leukemia
Source: Oncotarget. 2017 Dec 17;9(3):3853–66. doi: 10.18632/oncotarget.23380 (PMC5790505; doi:10.18632/oncotarget.23380)
Supplement: Supplementary file 1 [file oncotarget-09-3853-s001.pdf]

Identification of survivin as a promising target for the immunotherapy of adult B-cell acute lymphoblastic leukaemia

SUPPLEMENTARY MATERIALS

Supplementary Table 1: Cell lines used to demonstrate antigen expression (positive and negative controls) and optimise assays

Cell line	Disease type	Reference	Antigens expressed[†]	Method	Reference
697	Childhood ALL	[48]	Common ALL antigens & HLA-DR<br>END	 Flow cytometry	 [49]
ARH77	Non-Burkitt Lymphoblastoid cell line/myeloma	[50]	-	-	-
HeLa	Cervical cancer	[51]	PASD1	RT-PCR	[27]
H1299	Lung cancer	[52]	PASD1	RT-PCR, qPCR, ICC	[27],[53]
Jurkats	T-cell leukaemia	-	PASD1	RT-PCR	[27]
K562	Chronic myeloid leukaemia	[54]	HAGE<br>SSX2IP<br>SSX2<br>PASD1<br>WT1<br>Survivin	RT-PCR<br>RT-PCR, ICC<br>RT-PCR<br>RT-PCR, qPCR, ICC<br>RT-PCR<br>RT-PCR, Western Blot	[32]<br>[27], [55]<br>[56]<br>[27], [45]<br>[57]<br>[58]
KG1	Erythroleukaemia	[59]	*PASD1 and SSX2IP	RT-PCR	[27]
KYO-1	Chronic myeloid leukaemia	[60]			
MDA-MB-231*	Breast Adenocarcinoma	[61]	END	RT-PCR, Western blot, ICC	[13]
NB4	Acute promyelocytic leukaemia	[62]	Survivin	RT-PCR	[63]
OCI-LY3*	Non-Hodgkin's lymphoma	[64]	PASD1	ICC	[65]
P39	Myelomonocytoid	[66]	HAGE	RT-PCR	[32]
SW480	Colorectal cancer	[67]	WT1<br>PASD1	 cDNA hybridisation	[68]<br>[69]
U266	Multiple Myeloma	[70]	-	-	-
U937	Lymphoma	-	HAGE	RT-PCR	[32]
VLB	Leukaemic lymphoblasts	[71]	HAGE<br>SSX2IP	RT-PCR<br>RT-PCR	[32]<br>[27]

*- used only as controls for ICC analysis; †- that were also investigated in patients in this study; * - negative control for PASD1 and SSX2IP expression.

Supplementary Table 2: Antibodies used for ICC

Antibody specificity	Validation	Isotype	Manufacturer /catalogue number	Species	Dilution
**Primary antibodies**					
Actin	-	Monoclonal, IgG_1	Abcam/ab3280	Mouse anti-human	1/100
Isotype control	-	Monoclonal, IgG_1	Abcam/ab18443	Mouse anti-human	1/250
Isotype control	-	Monoclonal, IgG	Abcam/ab172730	Rabbit anti-human	1/250
pSSX2(N)	-	Polyclonal, IgG	Abcam/ab182361	Rabbit anti-human	1/100
WT1	[72]	Monoclonal, IgG_{2a}	Abcam/ab89901	Rabbit anti-human	1/50
SSX2IP	-	Polyclonal, IgG	Abcam/ab10256	Goat anti-human	1/100
Survivin	[73]	Monoclonal	Abcam/ab76424	Rabbit anti-human	1/100
PASD1-1: reacts with PASD1a and PASD1b [65]	[65]	N/A	Banham/Pulford University of Oxford	Mouse anti-human	1/250
Interlinking Ab for SSX2IP (GT175)	-	N/A	Banham/Pulford University of Oxford	Mouse anti-goat	1/100
**Secondary antibodies**					
Anti-mouse	-	Polyclonal, Ig	Dako#/K4007	Anti-mouse	N/A
Anti-rabbit	-	Polyclonal, Ig	Dako#/K4011	Anti-rabbit	N/A

N/A: not applicable. #: Part of the Dako Envision kit.
